# Decitabine Enhances Vγ9Vδ2 T Cell-Mediated Cytotoxic Effects on Osteosarcoma Cells *via* the NKG2DL–NKG2D Axis

**DOI:** 10.3389/fimmu.2018.01239

**Published:** 2018-06-01

**Authors:** Zhan Wang, Zenan Wang, Shu Li, Binghao Li, Lingling Sun, Hengyuan Li, Peng Lin, Shengdong Wang, Wangsiyuan Teng, Xingzhi Zhou, Zhaoming Ye

**Affiliations:** ^1^Centre for Orthopaedic Research, Orthopedics Research Institute of Zhejiang University, Department of Orthopaedics, The Second Affiliated Hospital of Zhejiang University School of Medicine, Hangzhou, China; ^2^Cancer Institute, Key Laboratory of Cancer Prevention and Intervention, Key Laboratory of Molecular Biology in Medical Sciences, National Ministry of Education, Department of Hematology, The Second Affiliated Hospital of Zhejiang University School of Medicine, Hangzhou, China

**Keywords:** decitabine, osteosarcoma, γδ T cell, MICB, UL16-binding protein 1

## Abstract

γδ T cell-based immunotherapy for osteosarcoma (OS) has shown limited success thus far. DNA-demethylating agents not only induce tumor cell death but also have an immunomodulatory function. In this study, we have assessed the potential benefit of combining decitabine (DAC, a DNA demethylation drug) and γδ T cells for OS immunotherapy. DAC increased the expression of natural killer group 2D (NKG2D) ligands (NKG2DLs), including major histocompatibility complex class I-related chains B (MICB) and UL16-binding protein 1 (ULBP1), on the OS cell surface, making the cells more sensitive to recognition and destruction by cytotoxic γδ T cells. The upregulation of MICB and ULBP1 was due to promoter DNA demethylation. Importantly, the killing of OS cells by γδ T cells was partially reversed by blocking the NKG2D receptor, suggesting that the γδ T cell-mediated cytolysis of DAC-pretreated OS cells was mainly dependent on the NKG2D–NKG2DL axis. The *in vivo* results were consistent with the *in vitro* results. In summary, DAC could upregulate MICB and ULBP1 expression in OS cells, and combination treatment involving γδ T cell immunotherapy and DAC could be used to enhance the cytotoxic killing of OS cells by γδ T cells.

## Introduction

Osteosarcoma (OS), which is the most common malignant primary bone tumor, predominantly affects children and young adults (1). The current standard treatment for OS includes preoperative neoadjuvant chemotherapy, surgical resection of the entire tumor, and postoperative chemotherapy (2). These treatment options have improved the 5-year survival of patients with localized OS to approximately 70%. However, the overall survival rate is ≤20% for patients with recurrence or distant metastases (3, 4). Moreover, since the 1980s, research on treatment for OS has fallen into stagnation (5). Therefore, there is an urgent need for a novel effective treatment for OS patients.

Although the combination of immunotherapy and other therapies is promising, T cell-based immunotherapy also presents challenges to those developing effective treatments for OS (6). In tumor microenvironments, γδ T cells act as an important innate immune cell population, mediating cytotoxic effects against a broad variety of neoplasms (7). Unlike αβ T cells, γδ T cells can distinguish tumor cells from normal cells and then kill them directly, without conventional major histocompatibility complex (MHC) restrictions (8). The antitumor effect of γδ T cells is induced by the interaction of γδ T cell receptors (TCRs) with tumor antigens. Additionally, other costimulatory receptors, such as the natural killer (NK) group 2D (NKG2D) receptor, expressed on γδ T cells can recognize NKG2D ligands (NKG2DLs), which can also induce the antitumor function of γδ T cells. These NKG2DLs on tumor cells include MHC class I-related chains A and B (MICA and MICB) and proteins in the UL16-binding protein family (ULBP1-6, mainly ULPBP1–3) (9). Clinical trials evaluating the use of γδ T cell immunotherapy for malignancies have confirmed its feasibility and acceptable safety profile (10–12). However, the effectiveness of γδ T cell-based immunotherapy for tumors is decreased because of its potential suppressive role in tumor microenvironments (13, 14).

Accumulating evidence has strongly indicated that γδ T cell immunotherapy for tumors could be combined with other therapies to improve clinical outcomes (6, 12, 14, 15). Hypomethylating agents such as 5-aza-2’-deoxycytidine [decitabine (DAC)] have been shown to have anticancer activity and additional immunomodulatory functions (16). Recently, several studies have reported that DAC-mediated hypomethylation restores NKG2DL expression in tumors and might, therefore, provide a clinically useful method for sensitizing tumors to γδ T cells (17, 18). However, the role of DAC in potentiating the antitumor effect of adoptive γδ T cell transfer remains unclear. We, therefore, investigated whether DAC could enhance the susceptibility of human OS cells to Vγ9Vδ2 T-cell-mediated cytotoxicity *via* the NKG2DL–NKG2D axis.

## Materials and Methods

### Cell Culture and Treatments

HOS and U2OS human OS cell lines were obtained from the Cell Collection of the Chinese Academy of Science (Shanghai, China). The HOS cells were cultured in Dulbecco’s Modified Eagle Medium (Gibco, Rockville, MD, USA) supplemented with 10% fetal bovine serum (Invitrogen, Carlsbad, CA, USA) and 1% penicillin (100 U/mL). The U2OS cells were cultured in Roswell Park Memorial Institute 1640 medium (Gibco) supplemented with 10% fetal bovine serum and 1% penicillin (100 U/mL). All cells were cultured at 37°C in the humid atmosphere of a 5% CO_2_ incubator. Cells were counted and transferred to plates or dishes 1 day prior to treatment, and the medium was then replaced with medium containing DAC (Sigma-Aldrich, St. Louis, MO, USA) at different concentrations. New medium containing DAC was added every day, and the cells were then harvested and counted for further experiments (19).

### *In Vitro* Expansion of Vγ9Vδ2 T Cells From Peripheral Blood Mononuclear Cells (PBMCs)

Peripheral blood samples were obtained from three healthy donors and the PBMCs were separated by density gradient centrifugation. The PBMCs were then seeded into 24-well culture plates. The detailed procedure for expanding human Vγ9Vδ2 T cells *in vitro* was described in our previous study (20). Negative magnetic-activated cell sorting (Miltenyi Biotec GmbH, Bergisch Gladbach, Germany) was used for purifying γδ T cells. Phenotypic analysis was performed by flow cytometry using anti-Vδ2-fluorescein isothiocyanate (cat. no. 331406, clone B6) and anti-CD3-allophycocyanin (cat. no. 300312, clone HIT3a) from BioLegend (San Diego, CA, USA).

The use of PBMCs was approved by the Ethics Committee of the Second Affiliated Hospital of Zhejiang University School of Medicine. Written informed consent was obtained from the three blood donors.

### Cytotoxicity Assay

An MTS assay was performed to analyze the cytotoxic effects of γδ T cells on OS cells *in vitro* (19). OS cells (HOS and U2OS cells) were plated in triplicate onto 96-well flat-bottomed plates at 2 × 10^3^, 5 × 10^3^, and 1 × 10^4^ cells/well for 72, 48, and 24 h of DAC pretreatment, respectively. γδ T cells were then added at the indicated effector/target (E:T) ratios and cocultured with OS cells for 4 h at 37°C in the humid atmosphere of a 5% CO_2_ incubator. Subsequently, all wells were gently washed with phosphate-buffered saline (PBS) twice to remove the γδ T cells. The percentage of viable cells was detected using an MTS assay (Promega, Madison, WI, USA). The absorbance was measured using a microplate reader at 490 nm.

To determine the cytotoxic pathways involved in γδ T cell-mediated cytolysis of OS cells, blocking experiments were carried out. In these experiments, γδ T cells were incubated with 10 µg/mL (saturating concentrations) of anti-human NKG2D (clone 149810; R&D Systems, Minneapolis, MN, USA), anti-pan-γδ TCR (clone B1; BD Biosciences, Franklin Lakes, NJ, USA), anti-human Fas ligand (FasL; clone NOK-2; BD Biosciences), or antitumor necrosis factor-related apoptosis-inducing ligand (Trail; clone RIK-2; BD Biosciences) for 30 min. The cells were then cocultured with OS cells to determine the cytotoxic pathways.

### Enzyme-Linked Immunosorbent Assay (ELISA)

γδ T cells were cocultured with untreated or DAC-pretreated OS cells in triplicate at an E:T ratio of 5:1. After 4 h, the supernatants were collected to assess the secretion of interferon (IFN)-γ using a human IFN-γ ELISA kit (Dakewe Biotech, Shenzhen, China).

### Reverse Transcription-Polymerase Chain Reaction

RNA was isolated using RNAiso Plus (TaKaRa Bio, Kusatsu, Japan) and subsequently transcribed into cDNA using a PrimeScript RT Reagent Kit (TaKaRa Bio). The mRNA was analyzed using a StepOnePlus Real-time PCR System (Applied Biosystems, Foster City, CA, USA) and SYBR Green (TaKaRa Bio). All primers used are listed in Table S2 in Supplementary Material. The amplification fold change was calculated using the 2^−ΔΔCt^ method.

### Flow Cytometry

For the analysis of apoptosis, target cells (γδ T cells) were stained with annexin V-phycoerythrin (PE) and 7-amino-actinomycin D (7-AAD) using a commercially available kit (BD Biosciences) according to the manufacturer’s instructions (21). The flow cytometry analysis of the target cells was performed on a FACSCanto flow cytometer (BD Biosciences).

The target cells were incubated with anti-human MICA, MICB, ULBP1, ULBP 2/5/6, or ULBP3 PE and its isotype control (R&D Systems) at 4°C. After 30 min, the cells were washed and analyzed using the FACSCanto flow cytometer. The mean fluorescence intensity (MFI) ratio was defined as the MFI of the specific staining relative to the MFI of the appropriate isotype control staining. Data analysis was performed using FlowJo vX software (TreeStar).

### Bisulfite Sequencing

HOS cells were treated with 5 µM DAC or PBS for 3 days. Bisulfite treatment was then used to extract genomic DNA from these cells. The MICB promoter was amplified with the following primers: 5′-TTGTTGTTTAGGTTGGAGTGTAGT-3′ (forward) and 5′-TATAATCCCAACACTTTAAAAAATC-3′ (reverse). The ULBP1 promoter was amplified with the following primers: 5′-TGTTTTTAGTGGAGAGGTAAAAAA-3′ (forward) and 5′-AAAAAACTTACATAAAAATACTCAATAAC-3′ (reverse). Each amplified product was cloned into a pUC18 T-vector and subsequently sent for DNA sequencing (Sangon Biotech, Shanghai, China) (22, 23).

### Western Blot Analysis

Osteosarcoma tumor tissues from mice were cut into pieces and lysed in radio immunoprecipitation assay lysis buffer to solubilize the proteins. The concentrations of protein extracts were determined using a bicinchoninic acid assay protein assay (Pierce, Rockford, IL, USA). An equal amount of protein was separated using 10% sodium dodecyl sulfate polyacrylamide gel electrophoresis (SDS-PAGE) and transferred to polyvinylidene difluoride membranes. The membranes were blocked using 5% nonfat milk and incubated with antibodies against glyceraldehyde 3-phosphate dehydrogenase (GAPDH; 1:1,000; Abcam, Cambridge, UK), MICB (1:1,000; Abcam), or ULBP1 (1:1,000; Abcam) overnight at 4°C. A horseradish peroxidase-conjugated secondary antibody was added for 1 h at room temperature. Immunoblots were assessed using enhanced chemiluminescence (Millipore, Burlington, MA, USA) and recorded using a Bio-Rad XRS chemiluminescence detection system (Bio-Rad, Hercules, CA, USA).

### *In Vivo* Experiments

Healthy 4-week-old female BALB/c-nu mice (which are immunodeficient) were purchased from the Shanghai SLAC Laboratory Animal Co., Ltd. (Shanghai, China). HOS cells (1 × 106 in 20 µL PBS) were engrafted by injecting them through the tibial plateau in the primary spongiosa of the left tibia.

The mice were randomly assigned to four treatment groups to confirm the efficacy of γδ T cells + DAC treatment, with each group containing three mice. Group 1 consisted of mice treated with PBS and served as the control, group 2 consisted of mice treated with γδ T cells and PBS, group 3 consisted of mice treated with DAC and PBS, and group 4 consisted of mice treated with both DAC and γδ T cells. The concentration of DAC was 1 µg/g of body weight, and it was dissolved in a final volume of 100 µL PBS (19). DAC and PBS were administered by intraperitoneal injection, whereas the *in vitro*-generated γδ T cells (5 × 10^7^ in 100 µL PBS) were administered by intravenous injection. The specific treatment plan is shown in Figure S1 in Supplementary Material. All mice were sacrificed at day 19 after treatment. The tumors were collected for measurement and weighing. The tumor volume (V) was estimated using the formula V = *a* × *b*^2^/2, where *a* is the maximum diameter of the tumor and *b* is the minimum diameter. The tumors were harvested for western blotting.

### Statistical Analysis

The data are presented as the mean ± SD. Differences between groups were estimated using the χ^2^ test or Student’s *t*-test. Statistical analysis was performed using Microsoft Excel 2016 (Microsoft, Redmond, WA, USA) and SPSS statistical software for Windows, version 21.0 (SPSS, Chicago, IL, USA). All tests of significance were two-tailed tests and significance was defined as *P* < 0.05.

## Results

### Effect of DAC on Apoptosis and Phenotype of Human γδ T Cells

**Figure 1:**
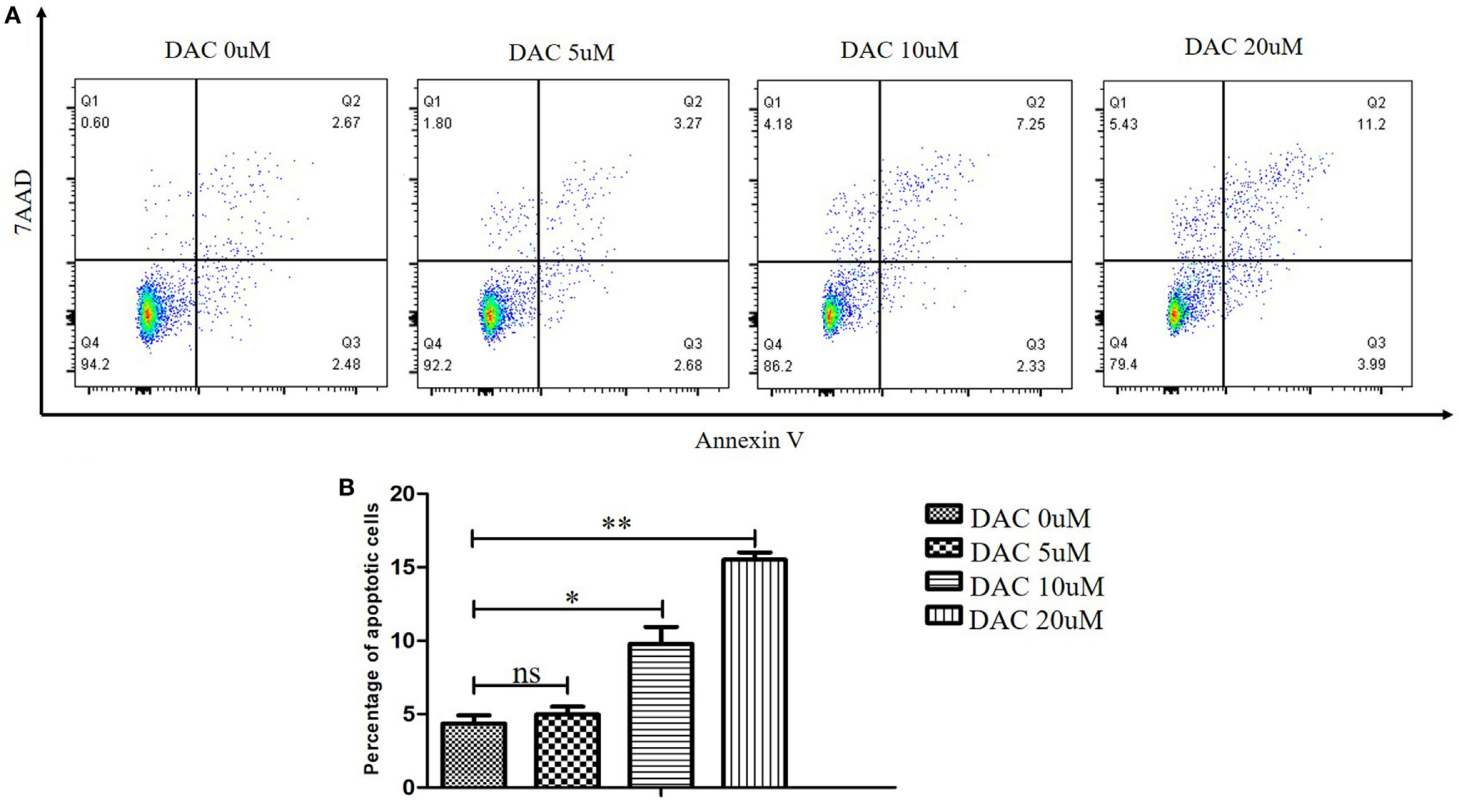
Effect of decitabine (DAC) on apoptosis in human γδ cells. DAC was added to γδ cell cultures at final concentrations ranging from 0 to 20 µM, or phosphate-buffered saline as vehicle control for 3 days. Media containing DAC was replenished every day. **(A)** Cell apoptosis was analyzed by flow cytometry with annexin V-PE/7-amino-actinomycin D (7-AAD). **(B)** The apoptotic rate was shown for three separate experiments. Error bars show SD among the replicates (*P < 0.05; **P < 0.01).

To assess the possible toxic effect of DAC when treating human γδ T cells, we performed cell apoptosis assays and analyzed the results using flow cytometry with annexin V-PE/7-AAD. DAC was added to γδ T cell cultures at 0, 5, 10, and 20 µM for 3 days. The media containing DAC were replenished every day. When DAC was used at 5 µM, no appreciable increase in the apoptosis of γδ T cells was observed (*P* > 0.05; Figure 1). Significantly increased apoptosis was detected when using ≥10 μM DAC. Subsequently, the DAC-induced expression of TCR and NKG2D in human γδ T cells was investigated using flow cytometry. Treatment with 5 µM DAC did not alter the percentage of γδ TCR^+^ and NKG2D^+^ γδ T cells (Figure S2 in Supplementary Material).

### Cytotoxic Effects of γδ T Cells on DAC-Pretreated HOS and U2OS Cell Lines

**Figure 2:**
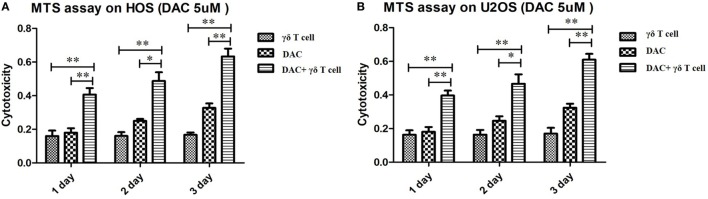
Cytotoxic assay using MTS method to evaluate the cytotoxic effects of γδ T cells on osteosarcoma (OS) cells pretreated with decitabine (DAC) (5 µM). **(A)** MTS assay on HOS cells. Treatment with γδ T cells or DAC alone slightly inhibited the growth of HOS cells. However, γδ T cells showed more efficiency in combination with DAC pretreatment for 1, 2, and 3 days. **(B)** MTS assay on U2OS cells. Similar to the results of HOS cells, DAC treatment can significantly enhance γδ T cells immunotherapy against OS cells. For **(A)** and **(B)**, samples were run in triplicates. Error bars show standard deviation among the replicates. (γδ T cell: tumor cell = 5:1; *P < 0.05; **P < 0.01).

To evaluate whether DAC treatment increased the susceptibility of OS cells to γδ T cell-mediated cytotoxicity, we performed MTS assays to evaluate the cytotoxic effects of γδ T cells on DAC-pretreated OS cell lines. As shown in Figure 2, treatment with γδ T cells or DAC alone slightly inhibited the proliferation of HOS and U2OS cells. However, γδ T cells in combination with DAC pretreatment for 24, 48, and 72 h exhibited significantly increased efficacy, indicating that DAC enhanced the susceptibility of OS cells to γδ T cell-mediated cytotoxicity.

### Upregulation of MICB and ULBP1 in HOS and U2OS Cell Lines After DAC Pretreatment

**Figure 3:**
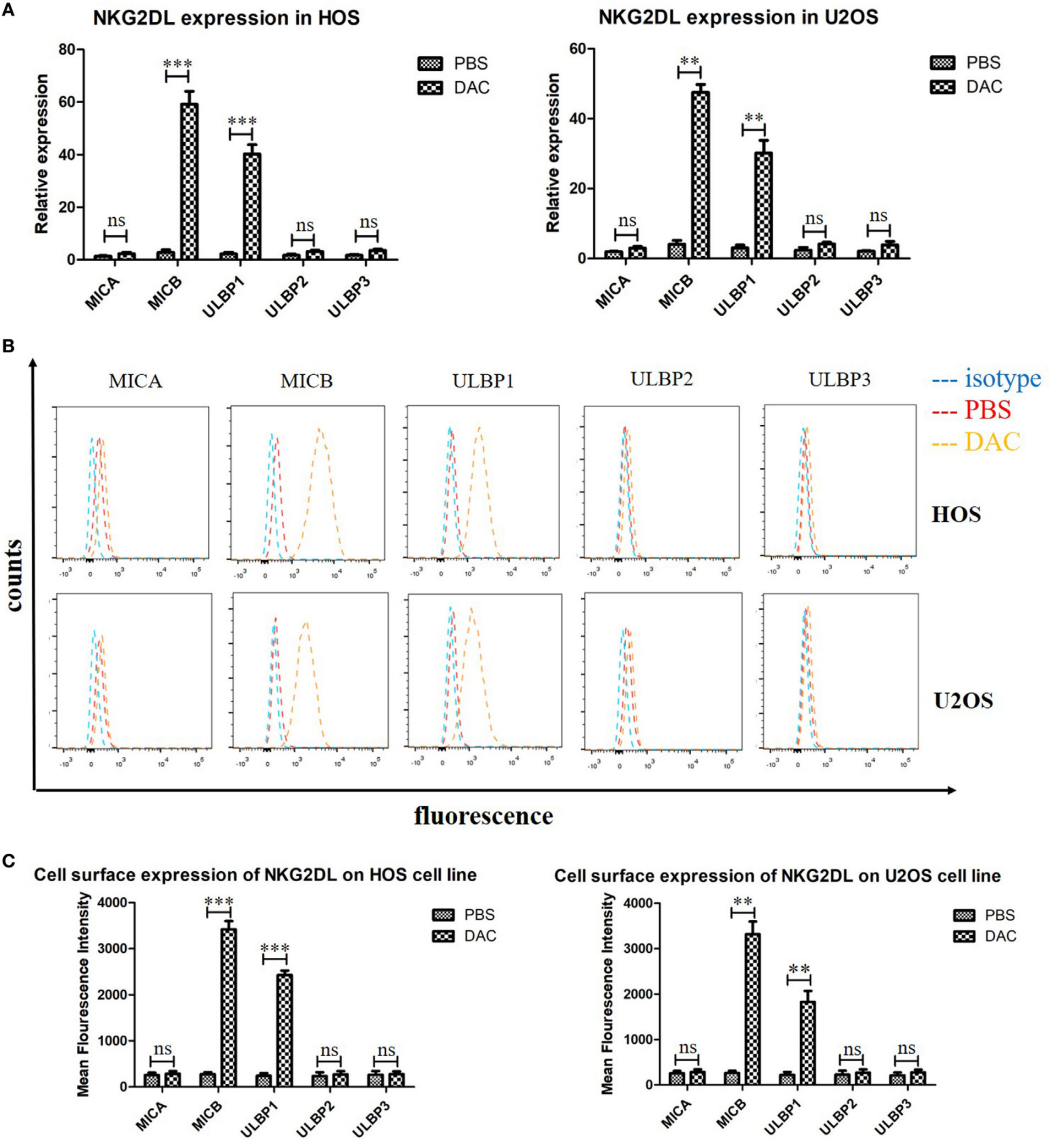
Increased expression of MICB and UL16-binding protein 1 (ULBP1) on osteosarcoma (OS) cells after 5 µM decitabine (DAC) treatment for 3 days. **(A)** NKG2DLs expression of HOS and U2OS cell lines were analyzed by reverse transcription-polymerase chain reaction. Expression levels are relative to GAPDH. MICB and ULBP1 expression was significantly increased after DAC treatment. While the rest NKG2DLs represented no obvious enhanced expression after DAC treatment. **(B,C)** Cell-surface expression of NKG2DL in OS cells was measured by flow cytometry and quantified by mean fluorescence intensity. The results were in line with PCR results. For **(A,C)**, samples were run in triplicates. Error bars show standard deviation among the replicates (ns refers to no significance. ***P < 0.001).

Previous studies have reported the role of the NKG2DL–NKG2D axis in the lysis of tumor cells by NK and γδ T cells (9, 24). Moreover, DAC treatment significantly increased NKG2DL expression in patients with acute myeloid leukemia (17). Whether DAC also increases NKG2DL expression in OS cells has not been previously explored. We assessed the cell-surface expression of NKG2DL in OS cells by PCR and flow cytometry. We found moderate to high levels of expression, with fivefold to >10-fold increases in the expression of the NKG2DLs MICB and ULBP1 in DAC-pretreated OS cells compared with PBS-pretreated OS cells. The expression of other NKG2DLs did not change significantly with DAC pretreatment (Figures 3A,B). Furthermore, the PCR results also showed that the expression levels of MICB and ULBP1 were significantly increased (*P* < 0.001) in DAC-pretreated OS cells compared with PBS-pretreated OS cells (Figure 3C).

### DAC-Induced Demethylation at MICB and ULBP1 Promoter Sites

**Figure 4:**
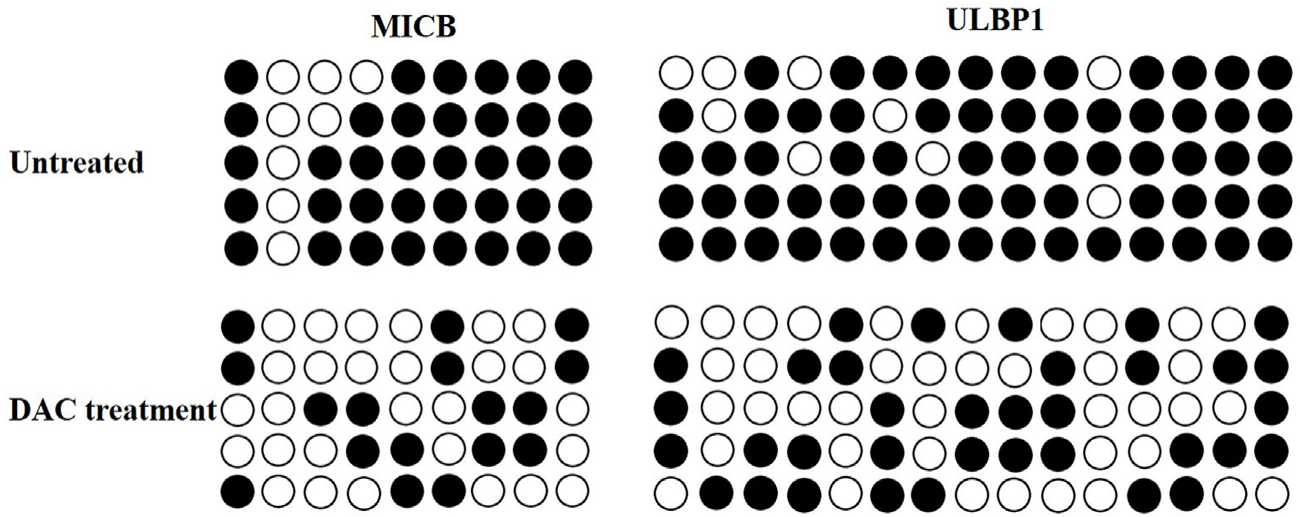
Decitabine (DAC) induces DNA demethylation at the MICB and UL16-binding protein 1 (ULBP1) promoter region. Cells were treated with 5 µM DAC for 3 days, and the methylation state of MICB and ULBP1 promoter was checked by bisulfite sequencing. Each circle represents a single CpG dinucleotide on the strand. Five clones from each cell line were analyzed. The open and filled circles represent the unmethylated and methylated CpG islands, respectively.

Decitabine treatment can activate methylated genes by promoter demethylation and the promotion of gene expression (25). Bisulfite sequencing revealed the high methylation levels of the MICB and ULBP1 promoters in untreated HOS cells, which were demethylated following DAC treatment (Figure 4). These results suggested that the upregulation of MICB and ULBP1 induced by DAC was associated with DNA demethylation.

### Recognition and Targeting of DAC-Pretreated OS Cells by γδ T Cells *via* NKG2D–NKG2DL Interactions *In Vitro*

**Figure 5:**
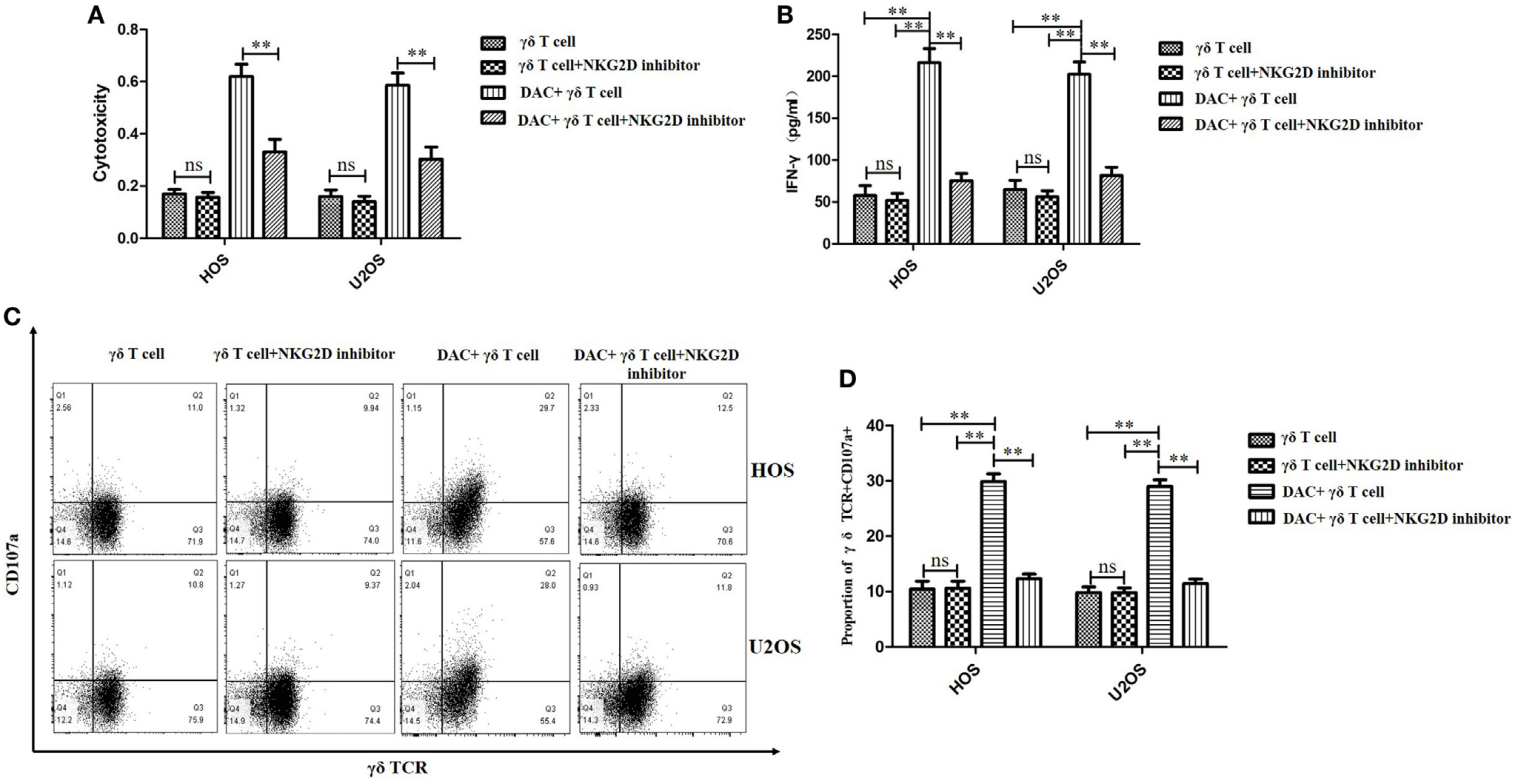
Decitabine (DAC) enhanced susceptibility of osteosarcoma (OS) cells to γδ T cell-mediated cytotoxicity *via* NKG2DL–natural killer group 2D (NKG2D) axis. Expanded γδ T cells were cocultured with OS cells for 4 h at an effector/target ratio of 5:1. **(A)** γδ T cells’ cytotoxicity on OS cells pretreated with DAC (5 µM) or phosphate-buffered saline for 3 days in the presence or absence of anti-NKG2D blocking antibodies (10 µg/mL) was determined by MTT assays. **(B)** Supernatants of **(A)** were harvested and assayed for interferon (IFN)-γ using a IFN-γ enzyme-linked immunosorbent assay kit. **(C,D)** Cell-surface expression of CD107a on γδ T cells was measured by flow cytometry and quantified by percent of γδ TCR^+^ CD107a^+^ T cells. For **(A,B,D)**, samples were run in triplicates. Error bars show standard deviation among the replicates (γδ T cell: tumor cell = 5:1; ns refers to no significance; **P < 0.01).

As DAC upregulated NKG2DL expression in OS cells, we speculated that γδ T cells recognize and target DAC-pretreated OS cells *via* NKG2D–NKG2DL interactions. To explore the molecular mechanisms involved in the interaction between γδ T cells and DAC-pretreated OS cells, a blocking experiment was performed to examine the effect of surface molecules on γδ T cell cytotoxicity. NKG2D blocking significantly reduced the cytotoxicity of γδ T cells against the DAC-pretreated OS cells (*P* < 0.01; Figure 5A). However, as shown in Figure 5A, NKG2D blocking had a weak effect on the cytotoxicity of γδ T cells against untreated OS cells.

**Figure 6:**
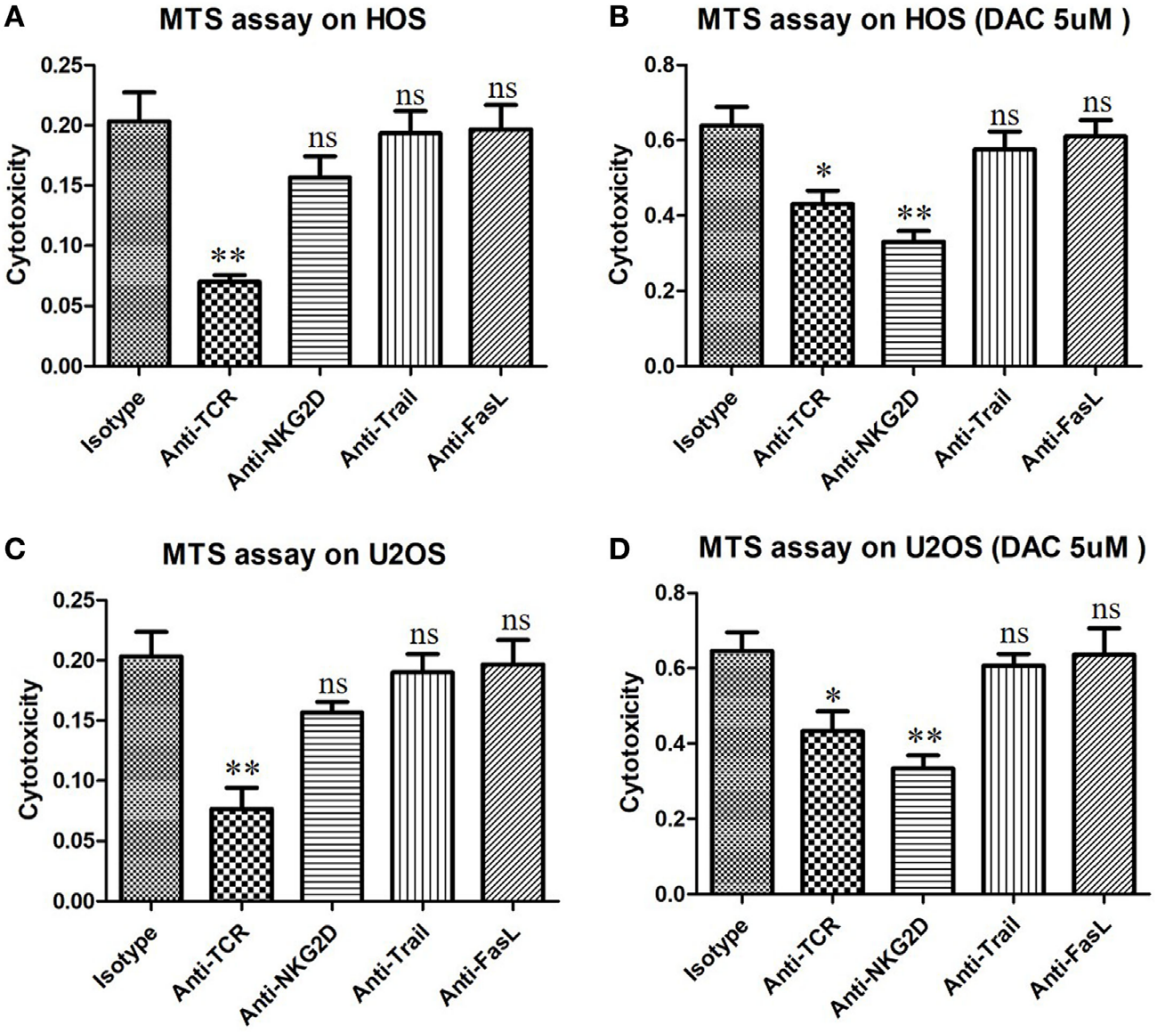
γδ T cells kill DAC-sensitized osteosarcoma (OS) cells mainly through the natural killer group 2D (NKG2D)–NKG2DL pathway. OS cells were pretreated with phosphate-buffered saline or decitabine (DAC) (5 µM) for 3 days. Then, γδ T cells were cocultured with DAC-pretreated OS cells for 4 h at an effector/target ratio of 5:1. Cytotoxicity of γδ T cells was determined using an MTS assay. γδ T cells were preincubated for 30 min with 10 µg/mL anti-human NKG2D, anti-pan-γδ T cell receptor (TCR), anti-Trail, or anti-FasL mAbs. Normally, TCR signal pathway was the key factor that can trigger the anti-OS activity of γδ T cells **(A,B)**. However, for DAC-pretreated OS cells, NKG2D–NKG2DL axis was the most important pathway by which γδ T cells killing about half of them **(C,D)**. Error bars show standard deviation among the replicates (*P < 0.05 vs. isotype control; **P < 0.01 vs. isotype control; ns, no significance vs. isotype control. mAb, monoclonal antibody).

Natural killer group 2D blocking significantly decreased the secretion of IFN-γ by γδ T cells when the OS cells were pretreated with DAC, but it had little effect on untreated OS cells (*P* < 0.01; Figure 5B). Moreover, the expression of CD107a was significantly increased during the γδ T cell killing of DAC-pretreated OS cells, and this expression was inhibited by blocking NKG2D. However, no obvious decrease in CD107a expression was observed during the γδ T cell killing of untreated OS cells after NKG2D blocking (*P* < 0.01; Figures 5C,D). Additionally, the cytotoxicity of the DAC + γδ T cells + NKG2D blocker group was significantly higher than that of the γδ T cells + NKG2D blocker group (*P* < 0.05). However, regarding IFN-γ secretion and CD107a expression, no significance was observed between these two groups. Although the TCR signaling pathway played an important role in γδ T cell-mediated cytotoxicity against untreated OS cells, for DAC-pretreated OS cells, the NKG2D–NKG2DL axis was the most important pathway. Approximately half of the killing of DAC-pretreated OS cells mediated by γδ T cells involved the NKG2D–NKG2DL axis (Figure 6). Taken together, these results suggested that the γδ T cell-mediated cytotoxic effects against DAC-pretreated OS cells were accomplished mainly through the NKG2D–NKG2DL pathway.

### Antitumor Effect of Combining Adoptive γδ T Cells With DAC Treatment *In Vivo*

**Figure 7:**
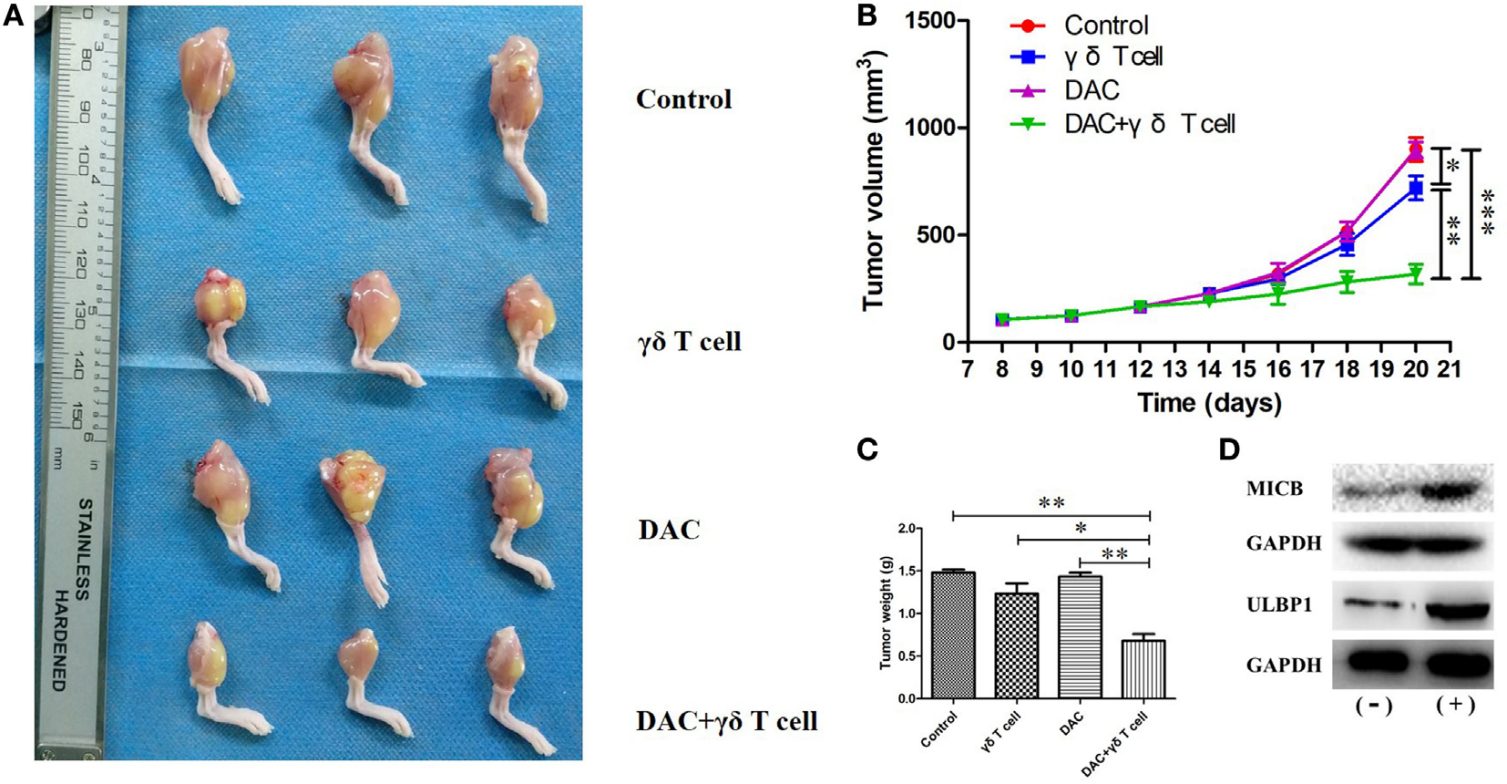
Mice receiving both γδ T cells and decitabine (DAC) showed significantly reduced tumor growth. **(A)** Mice from groups treated with γδ T cells + DAC showed less tumor burden than those mice untreated (control) or treated with DAC or γδ T cells alone. **(B)** Tumor volumes are significantly smaller in the group treated with γδ T cells + DAC compared with the control or DAC or γδ T cells group. **(C)** The group treated with γδ T cells + DAC showed significantly reduced tumor weight compared with the control or single-treatment group. Error bars show SD among the replicates. **(D)** Western blot confirmed that DAC treatment increased expression of MICB and UL16-binding protein 1 (ULBP1) *in vivo*. (−): without DAC treatment; (+): DAC treatment (ns refers to no significance; *P < 0.05; **P < 0.01; ***P < 0.001).

To confirm the effects of γδ T cells + DAC treatment *in vivo*, we created an OS model by injecting HOS cells into nude mice through the tibial plateau in the primary spongiosa of the left tibia. Mice in the control group were treated with PBS. As shown in Figure 7A, all mice in the control group showed progressive tumor growth. The mean tumor volume in the groups treated with DAC or γδ T cells alone was slightly less than that in the control group, whereas there was a substantial decrease in the tumor volume in mice receiving γδ T cells + DAC treatment compared to the control group (*P* < 0.01; Figure 7B). In addition, the tumor weight of the group receiving combined treatment was significantly reduced compared with that of the control or single-treatment groups (*P* < 0.05; Figure 7C). Additionally, western blotting showed that the MICB and ULBP1 proteins in OS tumor tissues increased after DAC treatment (Figure 7D). These results indicated that γδ T cells + DAC treatment markedly inhibited the growth of OS cells *in vivo*.

## Discussion

As a subpopulation of lymphocytes involved in the human innate immune system, γδ T cells can combat tumors and microbial infections. Unlike αβ T cells, γδ T cells can directly recognize and respond to antigens in an MHC-independent manner to effectively lyse tumor cells and microorganisms. Moreover, it is very difficult to achieve the required number of NK cells or αβ T cells *in vitro* to produce effective adoptive immunotherapy for tumors. In contrast, γδ T cells can be efficiently generated from PBMCs over a short period by *in vitro* culturing (26). Thus, γδ T cells have been suggested as a good candidate for cancer immunotherapy. Several clinical trials involving adoptive transfer of *in vitro*-expanded γδ T cells to control both hematological malignancies and solid tumors have been carried out, and they have shown promise regarding efficacy and safety (10–12, 27).

However, the efficacy of γδ T cell-based immunotherapy is limited because of its potential suppressive role in tumor microenvironments (13, 14). Regarding adoptive immunotherapy for treating tumors, human Vγ9Vδ2 T cells, which are the main γδ T cell population among PBMCs, are usually selected. However, Vδ2 T cells can exert immunosuppressive functions by producing suppressive cytokines, generating adenosine, upregulating negative costimulatory molecules, etc. They may also differentiate into cells with a regulatory phenotype similar to αβ Treg cells due to their high functional plasticity (28). Therefore, a better understanding of the functional role of Vδ2 T cells in tumor microenvironments will help to develop more effective antitumor immunotherapy.

Recently, the use of adoptive γδ T cell transfer in OS immunotherapy has become increasingly popular. In 2001, Kato et al. (29) documented the cytotoxicity of γδ T cells against OS cells, suggesting their potential use as anti-OS therapy. Preclinical studies have demonstrated the potential of using γδ T cells for OS treatment (30, 31). Although adoptive γδ T cell transfer alone has yielded promising clinical results, there is a strong rationale (based on preclinical research) for identifying the optimal combination treatment strategy to augment its efficacy (6, 32, 33).

Previous studies have shown that the cytotoxicity of γδ T cells against OS cells can be enhanced by other agents, such as trastuzumab (antihuman epidermal growth factor receptor 2 monoclonal antibody) and IFN-γ (20, 34). In addition, epigenetic modulators have recently been recognized as useful agents in cancer immunotherapy, especially γδ T cell-based immunotherapy (15). Hypomethylating agents, such as DAC, have been shown to have anticancer and immunomodulatory activities (16). Al-Romaih et al. (35) found that DAC-induced demethylation of growth arrest and DNA damage inducible alpha(GADD45A)and promoted apoptosis of OS cells. Zhang et al. (36) recently reported that DAC increased the levels of miR-370, an anti-oncogenic microRNA, resulting in the inhibition of OS cells. Regarding its mechanism of action in sarcoma immunotherapy, DAC may induce the upregulation of cancer/testis antigens, MHC molecules, and intracellular cell adhesion molecule-1 (ICAM-1) to promote immune recognition of sarcoma cells (37). Li et al. (19) reported that DAC treatment resulted in a CD8^+^ T cell-mediated anti-OS reaction *via* increasing expression of the MAGE-A family proteins and NY-ESO-1 in OS cells, both *in vitro* and *in vivo*, which has also been reported in other tumor cells (38, 39). A phase I trial combining DAC/dendritic cell vaccine was performed in two sarcoma patients, showing that this chemoimmunotherapeutic method was feasible and well tolerated, and providing a scientific basis for further studies (40).

Previous studies have focused on DAC treatment combined with adaptive immune cells but combinations involving innate immune cells are rare. Recent studies have reported that DAC-mediated hypomethylation restored NKG2DL expression in tumors and made tumor cells more sensitive to recognition and killing by NK cells (17, 18), indicating that the combined effect of DAC with the adoptive transfer of innate immune cells may be considerable. However, the combined effect of DAC with γδ T cells has not been previously explored. Thus, the purpose of our investigation was to demonstrate the effectiveness of this combination therapy for OS. DAC (5 µM) had no apparent cytotoxic effect on γδ T cells and did not alter the percentage of γδ TCR^+^ and NKG2D^+^ γδ T cells (Figure S2 in Supplementary Material). Cruz et al. (41) also found that DAC had minimal effects on the phenotype and function of T cells. In addition, Yang et al. (42) reported that treating tumor cells with DAC upregulated gene expression in a dose-dependent manner. Thus, in our study, 5 µM DAC was chosen for the subsequent experiments.

γδ T cells possess broad innate antitumor and anti-infective activities, which involve directly recognizing unprocessed phosphoantigens (PAgs) *via* cell-surface TCRs. In bacterial cells, PAgs are produced in the 1-deoxy-d-xylulose-5-phosphate pathway, whereas, in tumor cells, they are produced in the mevalonate pathway. Isopentenyl pyrophosphate (IPP) is one of the stress-induced PAgs in tumor cells, which can accumulate due to the inhibition of farnesyl pyrophosphate synthase (FPPS). FPPS is an enzyme that acts downstream of IPP in the mevalonate pathway. Moreover, inhibition of FPPS decreases the degree of prenylation, resulting in the accumulation of unprenylated Ras and Rap1A (43–45). Butyrophilin 3A (BTN3A, CD277) was recently identified as a critical mediator between PAgs and γδ T cells (46). BTN3A enhances γδ T cell-based immunotherapy for both hematological and solid tumors and has also been recognized as a beneficial prognostic marker in pancreatic ductal adenocarcinoma (47–49). However, precisely which tumor cell molecules are recognized by γδ TCRs remains unknown (46). In addition to the γδ TCR stimulus, the interactions involving NKG2D/NKG2DL, Trail/Trail-R, and Fas/FasL also activate the effector function of γδ T cells against tumors (26, 50).

Tumor cells express NKG2DL, which is a ligand of the NK cell-activating receptor NKG2D, on their cell surface. The interaction between NKG2D and NKG2DL triggers the cytolysis of tumor cells, such as OS cells (24). The NKG2D receptor is also expressed on the surface of γδ T cells, and γδ T cells have the same cytotoxic effect on tumor cells as NK cells. In the present study, we showed that γδ T cells exhibited significant efficacy when used in combination with DAC, suggesting that DAC may enhance the susceptibility of OS cells to γδ T cell-mediated cytotoxicity. Using PCR and flow cytometry, we then showed that DAC increased the expression of NKG2D ligands, primarily MICB and ULBP1, making OS cells more sensitive to recognition and destruction by cytotoxic γδ T cells. This effect of γδ T cells + DAC treatment may be unique to OS cells, because Zhang et al. (18) reported that DAC increased ULBP1 and ULBP3 expression in glioma cells and increased NK-mediated lysis of these cells in an NKG2D-dependent manner. In other words, NKG2DL expression may vary in different tumors after DAC treatment.

In the present study, DAC-induced demethylation of the promoter sites of MICB and ULBP1 in OS cells and promoted their expression, which was also reported by Tang et al. (23). Trail and FasL blocking had no obvious influence on the γδ T cell-mediated cytotoxic effect on OS cells regardless of DAC pretreatment (Figure 6). Although γδ T cell-mediated cytotoxicity against untreated OS cells occurred primarily in a TCR-dependent manner (Figures 6A,C), this mechanism was secondary (to a mechanism involving the NKG2D–NKG2DL axis) in DAC-pretreated OS cells (Figures 6B,D). Approximately half of the γδ T cell-mediated killing of the DAC-pretreated OS cells was reversed by blocking the NKG2D receptor. This suggested that the NKG2D–NKG2DL pathway played an important role during the γδ T cell-mediated cytolysis of DAC-pretreated OS cells. In the *in vivo* experiments in mice, no distinct side effects were observed. More importantly, we showed that combined γδ T cells + DAC treatment markedly inhibited the growth of OS, which provides a preclinical rationale for the use of DAC to sensitize OS to γδ T cell-mediated killing.

The present study has several limitations. First, the γδ T cells used in our study were all from healthy donors. Second, only HOS and U2OS cells were used in our study, and research with other human or murine OS cell lines and primary OS cells is required to verify the results of this study. Third, immunodeficient mice were used for the experiments with human OS cell lines, which limited the assessment of any additional effects of other adaptive immune cells (e.g., αβ T or B cells) in tumor microenvironments.

In summary, the results of the present study confirmed that DAC sensitizes OS cells to γδ T cell cytotoxicity. Furthermore, we demonstrated that the killing capacity of γδ T cells was potentiated by increasing the NKG2DL expression in DAC-pretreated OS cells. Adoptive transfer of γδ T cells combined with DAC treatment may represent a novel strategy for the treatment of OS and the findings of the present study provide evidence for the clinical application of this combination therapy.

## Ethics Statement

This study was approved by the Human and Animal Research Ethics Committee of the Second Affiliated Hospital of Zhejiang University School of Medicine. Written informed consent was obtained from all volunteers.

## Author Contributions

ZY and ZhanW designed research; ZhanW, ZenanW, and SL performed research; ZhanW, BL, LS, PL, SW, WT, and XZ analyzed data; ZY, ZhanW ZenanW, and SL wrote the manuscript.

## Conflict of Interest Statement

The authors declare that the research was conducted in the absence of any commercial or financial relationships that could be construed as a potential conflict of interest.

## Supplementary Material

Figure S1 Scheme of experimental groups and treatment procedure *in vivo*.

Figure S2 The human γδ T cells were identified by their expression of T cell receptor (TCR) and natural killer group 2D (NKG2D) after 5 µM decitabine (DAC) treatment for 1 day. The percentage of γδ TCR^+^ and NKG2D^+^ γδ T cells was analyzed by flow cytometry and no obvious change was observed between the two groups.
