# Supplementary figures and images for: Decitabine Enhances Vγ9Vδ2 T Cell-Mediated Cytotoxic Effects on Osteosarcoma Cells via the NKG2DL–NKG2D Axis
Source: Front Immunol. 2018 Jun 1;9:1239. doi: 10.3389/fimmu.2018.01239 (PMC5992281; doi:10.3389/fimmu.2018.01239)

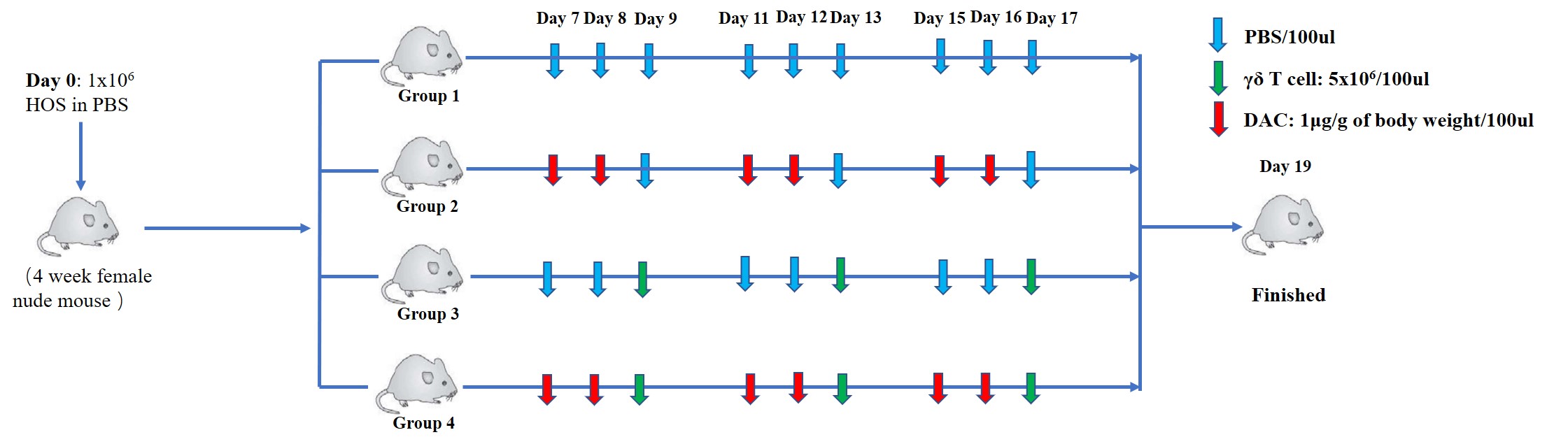

Supplement: Figure S1 — Scheme of experimental groups and treatment procedure in vivo. [file Image_1.jpeg]

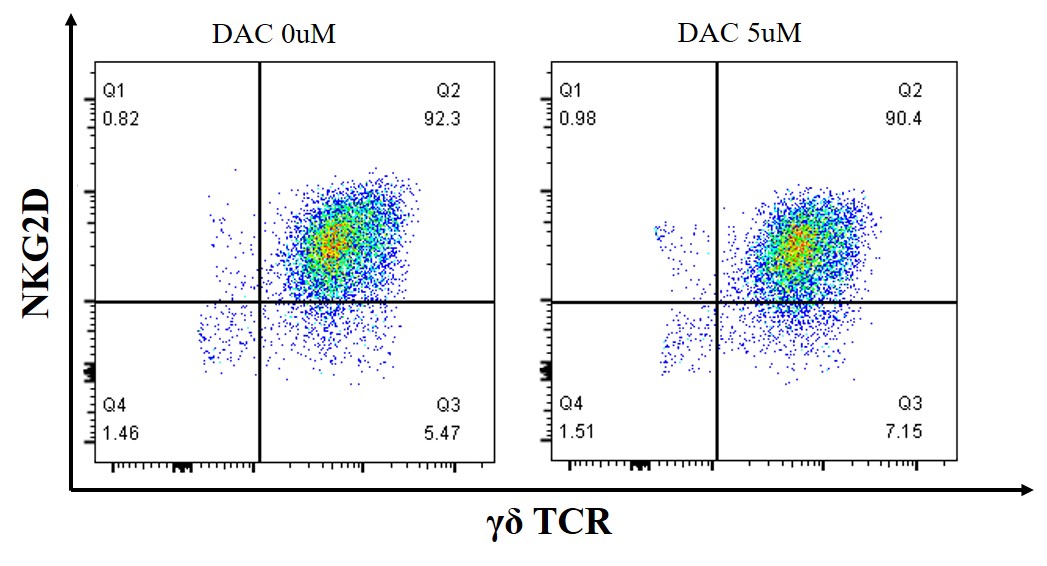

Supplement: Figure S2 — The human γδ T cells were identified by their expression of T cell receptor (TCR) and natural killer group 2D (NKG2D) after 5 µM decitabine (DAC) treatment for 1 day. The percentage of γδ TCR+ and NKG2D+ γδ T cells was analyzed by flow cytometry and no obvious change was observed between the two groups. [file Image_2.jpeg]
