# Supplementary material for: Decitabine Enhances Vγ9Vδ2 T Cell-Mediated Cytotoxic Effects on Osteosarcoma Cells via the NKG2DL–NKG2D Axis
Source: Front Immunol. 2018 Jun 1;9:1239. doi: 10.3389/fimmu.2018.01239 (PMC5992281; doi:10.3389/fimmu.2018.01239)
Supplement: Supplementary file 3 [file Table_1.docx]

Supplementary table 1: Primer sequences used in this study

| **Gene** | **Primer Sequence** |
| --- | --- |
| **GAPDH** | Forward: 5′-GAAGGTGAAGGTCGGAGTC-3′  Reverse: 5′-GAAGATGGTGATGGGATTTC-3′ |
| **MICA** | Forward: 5′- AGGGTTTCTTGCTGAGGTACA-3′  Reverse: 5′- GAGGTCCTTTCCGTTCCCTG-3′ |
| **MICB** | Forward: 5′- GCTTCAGAGTCAACGGACAGA-3′  Reverse: 5′- TGGAACCAGTGGACCCAGTA-3′ |
| **ULBP1** | Forward: 5′- TAAGTCCAGACCTGAACCACA-3′  Reverse: 5′- TCCACCACGTCTCTTAGTGTT-3′ |
| **ULBP2** | Forward: 5′- AGCAACTGCGTGACATTCAG-3′  Reverse: 5′- GCCATCCTATACAGTCTCCCA-3′ |
| **ULBP3** | Forward: 5′- TCTATGGGTCACCTAGAAGAGC-3′  Reverse: 5′- TCCACTGGGTGTGAAATCCTC-3′ |
